# Medical device biomaterials and antimicrobial resistance: exploring an overlooked research interface

**DOI:** 10.3389/fbioe.2026.1945426

**Published:** 2026-09-23

**Authors:** Pooja Sajish Valiparambil, Pascal Petit, Muhammad Asaduzzaman

**Affiliations:** 1 Usher Institute, University of Edinburgh, Edinburgh, United Kingdom; 2 Institute for Population Health, Weill Cornell Medicine, Doha, Qatar; 3 Independent Researcher, Environmental Epidemiologist, Grenoble, France; 4 Department of Community Medicine and Global Health, Institute of Health and Society, Faculty of Medicine, University of Oslo, Oslo, Norway

**Keywords:** antimicrobial resiatance, biofilm formation, biomateials, DEHP (di-(2-ethylhexyl) phthalate), healthcare-associated infections (HAI), medical device (MD), plasticisers, PVC (polyvinyl chloride)

## 1 Introduction

Modern healthcare depends heavily on polymer-based medical devices (Asokan et al., 2026a). Among these, polyvinyl chloride (PVC) is widely used in single-use medical devices such as intravenous fluid bags, blood storage bags, urinary catheters, endotracheal tubes, and dialysis circuits. Over 6 decades, PVC has accounted for a substantial proportion of medical plastics worldwide, valued for its flexibility, durability, transparency, chemical resistance and relatively low cost (Chiell et al., 2013). However, in clinical practice, PVC-based medical devices are not compositionally or functionally uniform. Medical-grade PVC is formulated with different plasticisers (e.g., DEHP, TOTM, DINCH), stabilisers, and additives, and undergoes diverse manufacturing and sterilisation processes, surface treatment, geometry, fluid contact and duration of clinical use. Throughout this article, we therefore refer to specific PVC-based device types and formulations where possible and treat any generalised claims about “PVC” as provisional and device-dependent.

While these devices have transformed patient care, they are also central to the epidemiology of healthcare-associated infections (HAIs). Indwelling devices, including urinary and vascular catheters and devices used for respiratory support, can provide surfaces for microbial attachment and biofilm formation and are associated with important device-related infectious complications (Asokan et al., 2026b). These infections can prolong hospitalisation and necessitate antimicrobial treatment, placing device-associated infection at an important intersection between infection prevention and antimicrobial use. However, the established risk associated with device use should be distinguished from the question of whether the biomaterial itself independently modifies infection or AMR-related outcomes.

AMR has simultaneously emerged as one of the greatest threats to global health. An estimated 1.14 million deaths were attributable to bacterial AMR globally in 2021, with the burden projected to increase substantially over coming decades (Naghavi et al., 2024). In response, global efforts to combat AMR have expanded considerably, centered on antimicrobial stewardship, One Health surveillance, infection prevention and control. Within this broader agenda, however, the potential relevance of medical-device biomaterial characteristics to processes associated with AMR has received comparatively limited investigation. Biomaterials interact directly with microorganisms, host tissues and administered drugs, raising questions about whether material-specific properties could influence intermediate processes relevant to infection or antimicrobial exposure. Whether these interactions translate into clinically meaningful AMR outcomes remains uncertain.

In this article, we examine three potential pathways through which medical-device biomaterials, with particular attention to PVC, could intersect with processes relevant to AMR. We distinguish established material- and device-related phenomena from indirect or hypothesis-generating downstream links to AMR and identify the principal evidence gaps requiring further investigation. Rather than proposing PVC or other biomaterials as established determinants of AMR, we use these pathways as a framework for examining an underexplored research interface and for considering priorities for future microbiological, pharmacological and clinical research.

## 2 Potential pathways

### 2.1 Pathway 1: biofilm formation and potential dissemination of resistance determinants

Before considering biofilms as a potential pathway relevant to AMR, it is essential to distinguish resistance from tolerance and persistence. AMR refers to heritable genetic changes that enable microorganisms to grow or survive at antimicrobial concentrations that would inhibit susceptible strains, typically reflected as an increased minimum inhibitory concentration (MIC), through mechanisms including target alteration, enzymatic degradation of drugs, permeability alteration, and efflux pumps (Brauner et al., 2016). Antimicrobial tolerance allows bacterial populations to survive transient exposure to bactericidal antibiotics without an increase in MIC, whereas persistence describes the survival of a small phenotypic subpopulation that can resume growth once antimicrobial pressure is removed (Brauner et al., 2016; Ba et al., 2019). These distinctions are particularly important in biofilms, where enhanced survival during antimicrobial exposure does not necessarily represent heritable resistance.

Medical device surfaces can support microbial adhesion and biofilm development, providing a biologically plausible pathway through which biomaterial properties could influence processes relevant to AMR. Surface chemistry, hydrophobicity, roughness, conditioning films, flow conditions, device geometry, microorganism, and duration of use can all influence bacterial attachment and biofilm development (Li et al., 2025; Cai et al., 2019; Oh et al., 2018; Niedźwiadek et al., 2025). Comparative studies have, for example, reported greater bacterial adhesion to some hydrophobic materials, including PVC, silicone and stainless steel, than to hydrophilic surfaces such as glass. However, these effects are highly context-dependent and vary according to the microorganism, surface characteristics and experimental conditions (Cai et al., 2019; Oh et al., 2018). Current evidence therefore supports material-dependent differences in microbial adhesion but does not establish that PVC carries a greater biofilm- or AMR-related risk than alternative medical-device materials.

Evidence from environmental plastics further suggests that plastic-associated microbial communities can provide conditions favorable to microbial interactions relevant to AMR (Balta et al., 2025). However, environmental microplastics differ substantially from indwelling medical devices in host exposure, flow conditions, microbial ecology, dwell time and antimicrobial pressure, limiting direct extrapolation to clinical PVC devices (Balta et al., 2025; Gomes et al., 2025; Li et al., 2020; Zhou et al., 2023). Such evidence therefore supports biological plausibility but should not be interpreted as evidence of a PVC-specific clinical AMR effect.

Once established, biofilms can enhance bacterial survival during antimicrobial exposure through mechanisms including reduced antimicrobial penetration, altered metabolic activity, stress responses and the formation of persister cells (Flemming et al., 2025; Hall and Mah, 2017; Lewis, 2010). These mechanisms predominantly represent antimicrobial tolerance and persistence rather than heritable resistance. Nevertheless, enhanced survival within biofilms may contribute to persistent or recurrent device-associated infections and repeated or prolonged antimicrobial exposure. These distinct biofilm-associated survival mechanisms are summarized in Figure 1.

**FIGURE 1:**
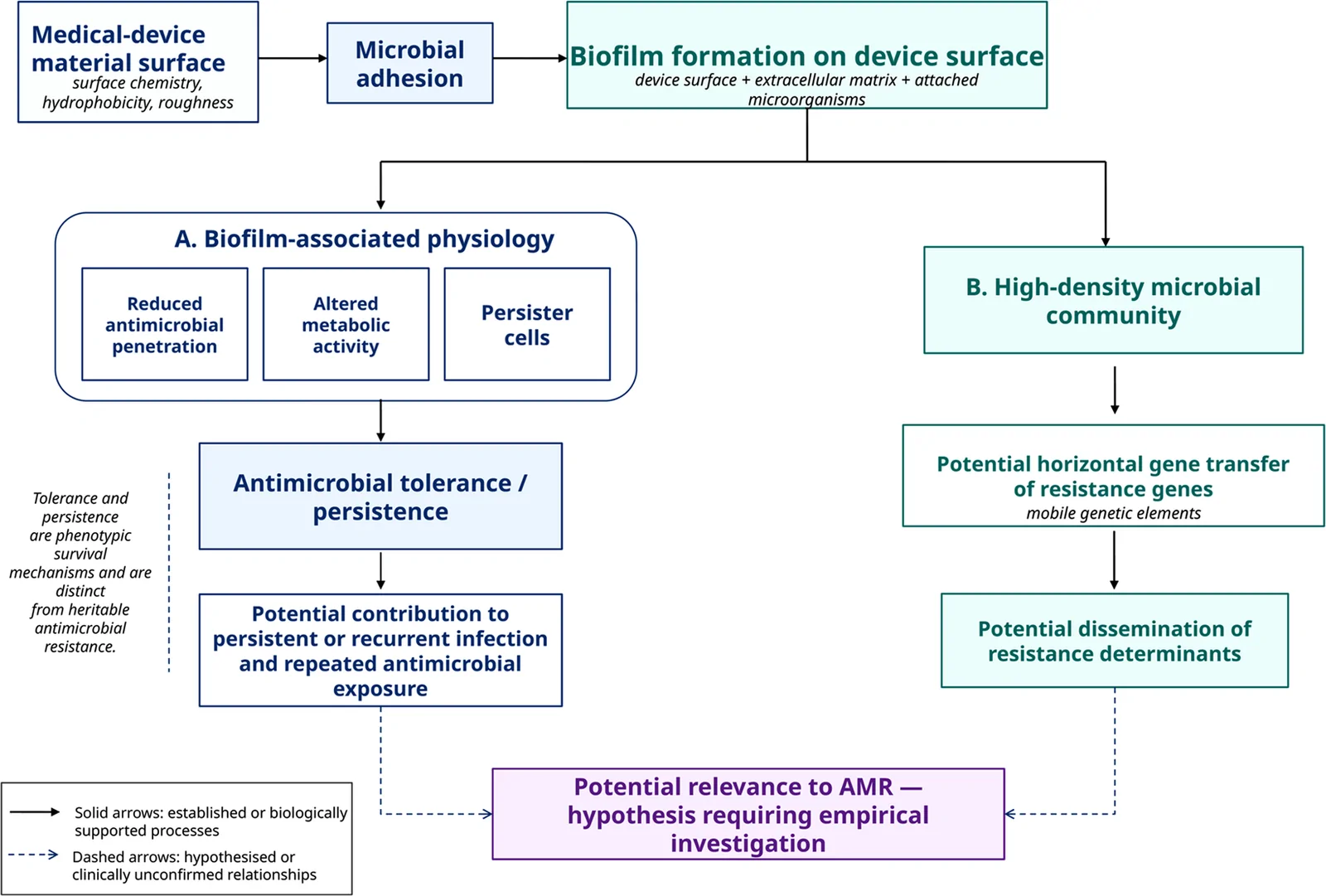
Proposed biofilm-associated pathway linking medical-device surfaces with processes potentially relevant to antimicrobial resistance.

A separate but related consideration is horizontal gene transfer (HGT). The close spatial organization, high cell density and extracellular matrix within biofilms can create conditions conducive to the transfer of mobile genetic elements, including those carrying antimicrobial resistance genes (Michaelis and Grohmann, 2023). However, this evidence relates to HGT within biofilms generally and does not establish a PVC-specific effect. There is currently no direct comparative clinical evidence demonstrating that PVC-based medical devices increase HGT relative to alternative biomaterials or that PVC-associated biofilms result in increased clinical AMR. HGT therefore represents a biologically plausible link between device-associated biofilms and the dissemination of resistance determinants, but its specific contribution within PVC medical devices remains unconfirmed.

A further hypothesis-generating consideration is co-selection between AMR and resistance to other selective agents. Environmental studies have demonstrated associations between metal resistance and antibiotic resistance genes, including co-occurrence on mobile genetic elements, while experimental evidence from microplastic-associated biofilms suggests that combined antibiotic and metal pressures can influence the abundance and co-selection of resistance determinants (Baker-Austin et al., 2006; Gillieatt and Coleman, 2024; Liu et al., 2022). However, these findings derive predominantly from environmental systems and cannot be directly extrapolated to medical-device biofilms. Whether comparable co-selection processes occur on PVC or other device materials *in vivo*, and whether they influence clinically relevant AMR, remains unknown.

Overall, the evidence is strongest for the ability of medical-device surfaces to support biofilm formation and for established biofilm-associated tolerance and persistence. Evidence linking these processes specifically to PVC is more limited, while the downstream progression from PVC-associated biofilm formation to HGT and clinically relevant AMR remains indirect and hypothesis-generating. Rather than directly generating resistance, PVC and other device biomaterials may provide microenvironments in which processes relevant to antimicrobial survival and the dissemination of resistance determinants can occur. Whether these effects differ meaningfully between biomaterials and translate into clinical AMR outcomes remains an important research gap.

### 2.2 Pathway 2: plasticiser leaching and potential immune-mediated effects

A second, more indirect pathway concerns plasticisers used in flexible PVC-based medical devices. Di(2-Ethylhexyl) Phthalate or DEHP has historically been one of the most widely used plasticisers in PVC medical products, although alternative plasticisers are increasingly used in some devices and regions. Because DEHP is not covalently bonded to the PVC polymer, it can migrate from medical devices into blood, intravenous fluids and other solutions in contact with the device (Tickner et al., 2001). The extent of migration varies according to the device formulation and conditions of use, including the contacting fluid, flow rate and duration of exposure (Bernard et al., 2018). Studies of PVC-based infusion systems have also demonstrated migration of alternative plasticisers, with substantial variation according to plasticiser type and clinical-use conditions (Bernard et al., 2015).

Clinical biomonitoring studies provide evidence that the use of DEHP-containing medical devices can result in substantial patient exposure. Elevated concentrations of DEHP metabolites have been reported among adult intensive-care patients, particularly those undergoing interventions involving extensive contact with medical tubing and extracorporeal circuits (Huygh et al., 2015). Device-associated exposure has similarly been demonstrated in neonates and infants receiving intensive medical interventions, including respiratory support and cardiac surgery (Green et al., 2005; Jenkins et al., 2021; Eckert et al., 2020). These findings establish that DEHP can leach from PVC medical devices and that clinically relevant exposure can occur, but they do not establish that such exposure increases infection susceptibility or contributes to AMR.

Evidence for downstream immune effects is less direct. Experimental studies suggest that DEHP and its metabolites can modulate inflammatory and immune pathways, but much of this evidence derives from *in vitro* and animal models, limiting extrapolation to clinically meaningful impairment of host defence. More recently, a prospective study of critically ill patients with sepsis reported an association between higher iatrogenic DEHP exposure and reduced 28-day survival and identified monocyte-related immune dysregulation as a potential mechanism (Gao et al., 2026). However, this study involved patients with established sepsis and did not demonstrate that DEHP exposure increased susceptibility to infection, increased antimicrobial use or influenced AMR outcomes.

The proposed connection between plasticiser exposure and AMR should therefore be regarded as hypothesis-generating rather than established. The evidence supports the initial steps of the pathway—leaching from PVC devices, patient exposure and potential modulation of immune responses—but becomes increasingly indirect downstream. Whether device-derived plasticiser exposure meaningfully alters host defence, increases infection risk or antimicrobial exposure, and ultimately influences AMR has not been demonstrated clinically. Establishing these intermediate links is therefore necessary before an AMR-related effect can be attributed to plasticiser exposure from PVC medical devices.

### 2.3 Pathway 3: drug-material sorption and potential antimicrobial underexposure

A third potential pathway concerns interactions between infused drugs and PVC-based administration systems. Drug sorption encompasses both adsorption to the internal surface of tubing and absorption into the polymer matrix. The extent of these interactions depends on several factors, including drug physicochemical properties (e.g., lipophilicity, protein binding, charge), formulation (solvent, surfactants, excipients), tubing composition and plasticiser chemistry, and infusion parameters such as flow rate, contact time, concentration, and priming practices. Experimental studies demonstrate that drug loss can vary substantially according to the specific drug-material combination, with more lipophilic compounds generally showing greater sorption than hydrophilic compounds (Tokhadze et al., 2019; Jin et al., 2016; Jin et al., 2017; Millot et al., 2026).

Comparative studies illustrate this variability. Tokhadze and colleagues evaluated plasticised PVC and several alternative infusion-tubing materials under simulated clinical conditions and observed substantial material-dependent differences in drug loss (Tokhadze et al., 2019). Diazepam showed marked absorption into some PVC-based systems, whereas insulin loss was predominantly attributable to surface adsorption and paracetamol demonstrated little or no measurable sorption. Similarly, studies of diazepam and tacrolimus have reported greater sorption with some PVC tubing than with alternative materials, although the magnitude of the effect depended on the drug, tubing material and infusion conditions (Jin et al., 2016; Jin et al., 2017). These findings establish that PVC and other polymeric administration systems can alter delivery of selected drugs, but they do not demonstrate a general effect across all medications.

The relevance of this phenomenon to antimicrobial therapy is less well established. Much of the available PVC sorption literature concerns non-antimicrobial drugs, particularly lipophilic agents such as diazepam and tacrolimus. Evidence cannot therefore be directly extrapolated to antibiotics, many of which have different physicochemical properties and may exhibit minimal interaction with PVC administration systems. Available antimicrobial-specific studies likewise suggest that interactions are drug- and device-specific rather than universal. Consequently, evidence that PVC infusion systems systematically reduce antimicrobial delivery is currently insufficient.

Furthermore, demonstration of drug loss within an administration system does not necessarily indicate clinically important underexposure. Clinical relevance depends on the proportion of drug lost, the administered dose, infusion duration, the drug’s therapeutic index, pharmacokinetic variability, protein binding, clearance, and whether the resulting exposure falls below a validated pharmacodynamic target. Molecular modelling of drug interactions with plasticised PVC further supports the physicochemical plausibility of adsorption and absorption (Millot et al., 2026), while studies of medication delivery through other tubing-based systems similarly demonstrate that device materials can alter delivery of selected compounds (Alodat et al., 2026). However, these data do not establish clinically significant antimicrobial underexposure.

The proposed downstream connection with AMR is therefore hypothesis-generating. If a specific antimicrobial undergoes substantial sorption within a particular administration system, reduced drug delivery could theoretically impair pharmacokinetic/pharmacodynamic target attainment. Inadequate antimicrobial exposure is an established concern in antimicrobial therapy more broadly; however, direct evidence demonstrating that PVC-associated sorption causes antimicrobial treatment failure or selects for resistant organisms is currently lacking. Drug–material sorption should therefore be regarded as a drug- and device-specific pharmacokinetic consideration rather than an established mechanism through which PVC medical devices contribute to AMR. Studies directly comparing antimicrobial concentrations, pharmacokinetic target attainment and clinical outcomes across PVC and alternative administration systems are needed to determine whether this pathway has clinical relevance.

The current evidence, key uncertainties and research priorities across the three proposed pathways are summarised in Table 1.

**TABLE 1:** Current evidence, uncertainties and research priorities for the proposed pathways linking PVC and other medical-device biomaterials with processes relevant to antimicrobial resistance.

Potential pathway	Current evidence and key uncertainty	Nature of evidence	Research priority
Pathway 1 *Biofilm formation and potential dissemination of resistance determinants*	Device-associated biofilm formation is well established, and surface characteristics can influence microbial adhesion. Biofilms promote antimicrobial tolerance and persistence and can provide conditions conducive to horizontal gene transfer (HGT). Environmental studies also support the possibility of co-selection of antibiotic and metal resistance determinants, but these findings cannot be directly extrapolated to clinical devices. Comparative evidence demonstrating that PVC increases HGT, co-selection or clinical AMR relative to alternative biomaterials is lacking	Direct evidence: Device-associated biofilm formation and biofilm-associated tolerance/persistence. Supportive evidence: HGT within biofilms and material-dependent microbial adhesion. Hypothesis-generating: PVC-specific HGT or co-selection and downstream clinical AMR effects	Comparative studies across clinically relevant biomaterials under realistic device conditions, followed by clinical studies evaluating biofilm characteristics, resistance-gene dissemination, device-associated infection, antimicrobial exposure and AMR outcomes
Pathway 2 *Plasticiser leaching and potential immune-mediated effects*	Migration of DEHP and alternative plasticisers from some flexible PVC-based devices is documented and varies by formulation and use conditions. Biomonitoring studies confirm substantial DEHP exposure in some highly exposed patients. Experimental and emerging clinical evidence suggests potential immune effects, but whether device-derived exposure impairs host defence or increases infection susceptibility remains uncertain. No direct clinical evidence establishes a downstream effect on antimicrobial use or AMR.	Direct evidence: Plasticiser migration and patient exposure. Indirect/supportive evidence: Modulation of inflammatory and immune responses. Hypothesis-generating: Altered host defence, increased infection susceptibility, subsequent antimicrobial exposure and AMR.	Prospective, device-specific exposure-response studies linking quantified plasticiser exposure with immune function, infection incidence and antimicrobial-use, with AMR-related outcomes where appropriate
Pathway 3 *Drug–material sorption and potential antimicrobial underexposure*	Sorption is documented for selected drug–material combinations and varies according to drug properties, tubing composition, plasticiser chemistry and administration conditions. Much of the available evidence concerns non-antimicrobial drugs. Evidence that PVC-associated sorption produces clinically meaningful antimicrobial underexposure, treatment failure or resistance selection remains limited	Direct evidence: Sorption of selected drugs in PVC and other polymeric administration systems. Direct evidence remains limited: Clinically meaningful antimicrobial loss attributable to PVC has not been established. Hypothesis-generating: Impaired antimicrobial PK/PD target attainment, treatment failure and selection for AMR.	Drug- and device-specific studies measuring antimicrobial concentrations under clinically realistic administration conditions, followed by assessment of PK/PD target attainment, treatment outcomes and emergence of resistance

Evidence descriptors indicate the nature of evidence supporting individual steps within each proposed pathway and do not represent a formal quality-of-evidence grading system. “Direct evidence” refers to evidence directly demonstrating the stated phenomenon; “indirect/supportive evidence” refers to evidence supporting an intermediate mechanistic step without demonstrating the complete pathway; and “hypothesis-generating” refers to biologically plausible downstream relationships that have not been directly demonstrated. AMR, antimicrobial resistance; DEHP, di(2-ethylhexyl) phthalate; HGT, horizontal gene transfer; PK/PD, pharmacokinetic/pharmacodynamic; PVC, polyvinyl chloride.

## 3 Discussion

### 3.1 Policy implications and future research priorities

Global AMR policy has evolved considerably over the past decade. The WHO Global Action Plan on AMR (GAP-AMR) identifies five strategic objectives: awareness, surveillance, infection prevention, optimizing antibiotic use, and economic investment in new medicines (World Health Organization, 2015). The updated Global Action Plan on AMR 2026–2036 further strengthens the emphasis on coordinated One Health action, prevention, surveillance, responsible antimicrobial use, research and innovation (World Health Organization, 2026). Within these frameworks, medical-device biomaterials are not generally considered as distinct variables in AMR surveillance. This should not be interpreted as a deficiency in existing AMR policy, but rather as reflecting the limited evidence that material-specific differences translate into clinically meaningful AMR outcomes.

Medical-device regulation already incorporates risk-based assessment of material and device safety. In the United States, FDA premarket pathways, including the 510(k) process where applicable, consider device safety and performance, while biological evaluation of patient-contacting materials is undertaken within a risk-management framework informed by standards such as ISO 10993-1 (International Organization for Standardization, 2025). In the European Union, Regulation (EU) 2017/745 establishes requirements relating to the chemical, physical and biological properties of medical devices and the prevention or minimisation of infection and microbial contamination (European Parliament and Council of the European Union, 2017). ISO 14971:2019 similarly provides a lifecycle framework for identifying, evaluating, controlling and monitoring device-related risks (International Organization for Standardization, 2019). These frameworks therefore provide mechanisms through which material-related biological and infection risks can be considered. However, potential downstream AMR-relevant consequences of material–microbe, plasticiser–host and material–drug interactions are not currently established as distinct outcomes warranting routine comparative assessment across biomaterials.

The immediate priority should therefore be evidence generation rather than the introduction of new AMR-specific regulatory requirements. Comparative studies should evaluate clinically relevant device materials and formulations under realistic use conditions to determine whether material-specific differences influence biofilm formation, infection-related or antimicrobial-exposure outcomes. Consistent reporting of device composition, plasticisers and other relevant additives, surface characteristics, duration of use and clinical context would improve reproducibility and enable meaningful comparison across studies. Where clinically relevant material-specific differences are demonstrated, such evidence could subsequently inform risk assessment, health technology assessment and procurement decisions alongside established considerations of safety, performance, clinical benefit and cost.

Post-market evidence may also have an important role where material-specific safety signals emerge. Existing regulatory mechanisms provide opportunities for post-market monitoring and targeted investigation, although their scope and application vary by device and jurisdiction. Rather than assuming that new regulatory structures are required, emerging evidence on biomaterial-related infection or AMR-relevant outcomes could initially be evaluated within these existing risk-management and post-market frameworks. Additional regulatory requirements would be justified only if sufficiently robust evidence demonstrates clinically important risks that are not adequately addressed through existing mechanisms.

Research should also consider populations with high cumulative device exposure or increased susceptibility to adverse outcomes. Neonates and critically ill patients, for example, may experience prolonged or repeated exposure to intravenous tubing, extracorporeal circuits, ventilation or other medical devices. Neonates may additionally have immature physiological systems that influence responses to chemical exposures. These groups may therefore be particularly informative populations for studies examining device-derived plasticiser exposure, material-associated biofilm formation and drug–material interactions. However, vulnerability to one proposed pathway should not be assumed to imply vulnerability to all pathways, and population-specific risks should be evaluated empirically.

Overall, an incremental approach is warranted. Systematic documentation of device materials and formulations, comparative evaluation of clinically relevant biomaterials, and integration of material-specific variables into appropriately designed infection, pharmacological and post-market studies would help determine whether the pathways proposed here have measurable clinical consequences. Regulatory or procurement responses should follow, rather than precede, evidence demonstrating that particular biomaterials or formulations meaningfully influence infection, antimicrobial exposure or AMR-related outcomes.

### 3.2 Conclusion

Medical device biomaterials represent a potentially relevant but insufficiently investigated dimension of the broader AMR landscape. This article has examined three pathways through which properties of PVC and other device materials could intersect with processes relevant to AMR. Evidence supports several upstream components of these pathways, including device-associated biofilm formation, plasticiser migration and patient exposure, and drug–material interactions. However, evidence becomes increasingly indirect when these phenomena are linked to downstream AMR outcomes, and a causal relationship between PVC or other medical-device biomaterials and clinical AMR has not been established.

The priority is therefore not to attribute AMR risk to particular biomaterials prematurely, but to determine whether material-specific differences translate into clinically meaningful effects on infection, antimicrobial exposure or resistance-related outcomes. Comparative studies conducted under realistic conditions, together with more consistent reporting of device composition and formulation, will be essential to address this evidence gap. Integrating biomaterial characteristics into appropriately designed microbiological, pharmacological and clinical research could clarify whether medical-device materials represent a modifiable factor within the complex determinants of AMR and, if so, provide an evidence base for future infection-prevention, procurement or regulatory decisions.

## References

[B1] Alodat L. Shahin W. Breik L. Allahham A. (2026). Stability of medications administered *via* enteral feeding tubes: a systematic review. Clin. Transl. Sci. 19 (1), e70427. 10.1111/cts.70427 41443608 PMC12737946

[B2] Asokan S. Pandey R. K. Jalil M. A. Alhussen S. K. A. Yousif S. I. A. Abbas R. K. (2026a). Biofilm associated infections on medical devices: pathogenesis, diagnostic challenges, and control strategies. Microbe 11, 100712. 10.1016/j.microb.2026.100712

[B3] Asokan S. Banerjee N. Saleem M. Atiyah H. M. Pandey R. K. Abbas R. K. (2026b). Healthcare associated infections (HAI): insights into epidemiology, microbiology, and diagnostics. Diagnostic Microbiol. Infect. Dis. 115, 117376. 10.1016/j.diagmicrobio.2026.117376 41865593

[B4] Balaban N. Q. Helaine S. Lewis K. Ackermann M. Aldridge B. Andersson D. I. (2019). Definitions and guidelines for research on antibiotic persistence. Nat. Rev. Microbiol. 17 (7), 441–448. 10.1038/s41579-019-0196-3 30980069 PMC7136161

[B5] Baker-Austin C. Wright M. S. Stepanauskas R. McArthur J. (2006). Major uncertainties and future research opportunities in metal–antibiotic co-selection. Trends Microbiol. 14 (4), 176–182. 16537105 10.1016/j.tim.2006.02.006

[B6] Balta I. Lemon J. Gadaj A. Cretescu I. Stef D. Pet I. (2025). The interplay between antimicrobial resistance, heavy metal pollution, and the role of microplastics. Front. Microbiol. 16, 1550587. 10.3389/fmicb.2025.1550587 40092036 PMC11906687

[B7] Bernard L. Cueff R. Breysse C. Décaudin B. Sautou V. , and Armed Study Group (2015). Migrability of PVC plasticizers from medical devices into a simulant of infused solutions. Int. J. Pharm. 485 (1-2), 341–347. 10.1016/j.ijpharm.2015.03.030 25796128

[B8] Bernard L. Eljezi T. Clauson H. Lambert C. Bouattour Y. Chennell P. (2018). Effects of flow rate on the migration of different plasticizers from PVC infusion medical devices. PLOS ONE 13 (2), e0192369. 10.1371/journal.pone.0192369 29474357 PMC5825016

[B9] Brauner A. Fridman O. Gefen O. Balaban N. Q. (2016). Distinguishing between resistance, tolerance and persistence to antibiotic treatment. Nat. Rev. Microbiol. 14 (5), 320–330. 10.1038/nrmicro.2016.34 27080241

[B10] Cai L. Wu D. Xia J. Shi H. Kim H. (2019). Influence of physicochemical surface properties on the adhesion of bacteria onto four types of plastics. Sci. Total Environ. 671, 1101–1107. 10.1016/j.scitotenv.2019.03.434

[B11] Chiellini F. Ferri M. Morelli A. Dipaola L. Latini G. (2013). Perspectives on alternatives to phthalate plasticized poly(vinyl chloride) in medical devices applications. Prog. Polym. Sci. 38 (7), 1067–1088. 10.1016/j.progpolymsci.2013.03.001

[B12] Eckert E. Müller J. Höllerer C. Purbojo A. Cesnjevar R. Göen T. (2020). Plasticizer exposure of infants during cardiac surgery. Toxicol. Lett. 330, 7–13. 10.1016/j.toxlet.2020.04.004 32387387

[B13] European Parliament and Council of the European Union (2017). Regulation (EU) 2017/745 of the European Parliament and of the Council of 5 April 2017 on Medical Devices. Official Journal of the European Union, 1–175.

[B14] Flemming H.-C. van Hullebusch E. D. Little B. J. Neu T. R. Nielsen P. H. Seviour T. (2025). Microbial extracellular polymeric substances in the environment, technology and medicine. Nat. Rev. Microbiol. 23 (2), 87–105. 10.1038/s41579-024-01098-y 39333414

[B15] Gao J. J. Huang L. Z. Wang T. Zhang Y. L. Ye K. Lu W. H. (2026). Iatrogenic plasticizer Di(2-ethylhexyl) phthalate (DEHP) exposure increases sepsis mortality risk: machine learning implicates monocyte-driven immune dysregulation. Toxicol. Appl. Pharmacol. 509, 117752. 10.1016/j.taap.2026.117752 41651080

[B16] Gillieatt B. F. Coleman N. V. (2024). Unravelling the mechanisms of antibiotic and heavy metal resistance co-selection in environmental bacteria. FEMS Microbiol. Rev. 48 (4), fuae017. 10.1093/femsre/fuae017 38897736 PMC11253441

[B17] Gomes Y. Tavares A. Barbosa R. Tomaz A. Sousa W. Oliveira L. (2025). Biological responses to biomaterials: a review. Braz. J. Med. Biol. Res. 58, e14599. 10.1590/1414-431X2025e14599 40367018 PMC12068782

[B18] Green R. Hauser R. Calafat A. M. Weuve J. Schettler T. Ringer S. (2005). Use of di(2-ethylhexyl) phthalate-containing medical products and urinary levels of mono(2-ethylhexyl) phthalate in neonatal intensive care unit infants. Environ. Health Perspect. 113 (9), 1222–1225. 10.1289/ehp.7932 16140631 PMC1280405

[B19] Hall C. W. Mah T.-F. (2017). Molecular mechanisms of biofilm-based antibiotic resistance and tolerance in pathogenic bacteria. FEMS Microbiology Reviews 41 (3), 276–301. 10.1093/femsre/fux010 28369412

[B20] Huygh J. Clotman K. Malarvannan G. Covaci A. Schepens T. Verbrugghe W. (2015). Considerable exposure to the endocrine disrupting chemicals phthalates and bisphenol-A in intensive care unit (ICU) patients. Environ. Int. 81, 64–72. 10.1016/j.envint.2015.04.008 25955314

[B21] International Organization for Standardization (2019). ISO 14971:2019 Medical devices—Application of Risk Management to Medical Devices. Geneva: International Organization for Standardization.

[B22] International Organization for Standardization (2025). Biological Evaluation of Medical devices—Part 1: Requirements and General Principles for the Evaluation of Biological Safety Within a Risk Management Process. Geneva: International Organization for Standardization.

[B23] Jenkins R. Ondusko D. Montrose L. Forbush R. Rozansky D. (2021). Phthalate exposures in the neonatal intensive care unit. Toxics 9 (5), 90. 10.3390/toxics9050090 33919093 PMC8143182

[B24] Jin S.-E. You S. Jeon S. Hwang S.-J. (2016). Diazepam sorption to PVC-and non-PVC-based tubes in administration sets with quantitative determination using a high-performance liquid chromatographic method. Int. J. Pharm. 506 (1-2), 414–419. 10.1016/j.ijpharm.2016.04.040 27091292

[B25] Jin S.-E. Jeon S. Byon H.-J. Hwang S.-J. (2017). Evaluation of tacrolimus sorption to PVC-and non-PVC-based tubes in administration sets: pump method vs. drip method. Int. J. Pharm. 528 (1-2), 172–179. 10.1016/j.ijpharm.2017.05.040 28549974

[B26] Lewis K. (2010). Persister cells. Annu. Review Microbiology 64, 357–372. 10.1146/annurev.micro.112408.134306 20528688

[B27] Li C. Guo C. Fitzpatrick V. Ibrahim A. Zwierstra M. J. Hanna P. (2020). Design of biodegradable, implantable devices towards clinical translation. Nat. Rev. Mater. 5 (1), 61–81. 10.1038/s41578-019-0150-z

[B28] Li S. Bi C. Xiang B. Wang Z. Yang H. Fu C. (2025). The role of cyclic di-GMP in biomaterial-associated infections caused by commensal *Escherichia coli* . Plos One 20 (8), e0330229. 10.1371/journal.pone.0330229 40833985 PMC12367115

[B29] Liu X. Wang H. Li L. Deng C. Chen Y. Ding H. (2022). Do microplastic biofilms promote the evolution and co-selection of antibiotic and metal resistance genes and their associations with bacterial communities under antibiotic and metal pressures? J. Hazardous Materials 424, 127285. 10.1016/j.jhazmat.2021.127285 34597934

[B30] Michaelis C. Grohmann E. (2023). Horizontal gene transfer of antibiotic resistance genes in biofilms. Antibiotics 12 (2), 328. 10.3390/antibiotics12020328 36830238 PMC9952180

[B31] Millot M. S. Devémy J. Chennell P. Dequidt A. Sautou V. Sahihi M. (2026). Coarse-grained modeling of drug absorption into plasticized PVC. J. Chem. Theory Comput. 22 (5), 2501–2514. 10.1021/acs.jctc.5c02155 41729242

[B32] Naghavi M. Vollset S. E. Ikuta K. S. Swetschinski L. R. Gray A. P. Wool E. E. (2024). Global burden of bacterial antimicrobial resistance 1990-2021: a systematic analysis with forecasts to 2050. Lancet 404 (10459), 1199–1226. 10.1016/s0140-6736(24)01867-1 39299261 PMC11718157

[B33] Niedźwiadek K. Polak-Berecka M. Waśko A. (2025). Innovations in biofilm prevention and eradication in medical sector: an integrative review. Pathogens 14 (12), 1242. 10.3390/pathogens14121242 41471197 PMC12735767

[B34] Oh J. K. Yegin Y. Yang F. Zhang M. Li J. Huang S. (2018). The influence of surface chemistry on the kinetics and thermodynamics of bacterial adhesion. Sci. Reports 8 (1), 17247. 10.1038/s41598-018-35343-1 30467352 PMC6250697

[B35] Tickner J. A. Schettler T. Guidotti T. McCally M. Rossi M. (2001). Health risks posed by use of Di‐2‐ethylhexyl phthalate (DEHP) in PVC medical devices: a critical review. Am. Journal Industrial Medicine 39 (1), 100–111. 10.1002/1097-0274(200101)39:1<100::aid-ajim10>3.0.co;2-q 11148020

[B36] Tokhadze N. Chennell P. Bernard L. Lambert C. Pereira B. Mailhot-Jensen B. (2019). Impact of alternative materials to plasticized PVC infusion tubings on drug sorption and plasticizer release. Sci. Rep. 9 (1), 18917. 10.1038/s41598-019-55113-x 31831771 PMC6908714

[B37] World Health Organization (2015). Global Action Plan on Antimicrobial Resistance. Geneva: World Health Organization. Available online at: https://www.who.int/publications/i/item/9789241509763 (Accessed July 14, 2026).

[B38] World Health Organization (2026). Global Action Plan on Antimicrobial Resistance 2026–2036. Geneva: World Health Organization.

[B39] Zhou T. Wu J. Hu X. Cao Z. Yang B. Li Y. (2023). Microplastics released from disposable medical devices and their toxic responses in *Caenorhabditis elegans* . Environ. Res. 239, 117345. 10.1016/j.envres.2023.117345 37821065

